# The Objective Response and Disease Control Rates in Patients with Liver Metastastic Breast Cancer Receiving Transarterial Radioembolization: A Meta-Analysis

**DOI:** 10.3390/curroncol31110508

**Published:** 2024-11-03

**Authors:** Natale Quartuccio, Vincenzo Militano, Marco Pappalardo, Luca Filippi, Oreste Bagni, Antonino Maria Moreci, Salvatore Ialuna

**Affiliations:** 1 Nuclear Medicine Unit, Ospedali Riuniti Villa Sofia-Cervello, 90146 Palermo, Italy; n.quartuccio@villasofia.it (N.Q.);; 2 Nuclear Medicine Unit, Azienda Ospedaliera “Pugliese-Ciaccio”, 88100 Catanzaro, Italy; 3 Division of Plastic Surgery, Università degli Studi di Modena e Reggio Emilia, 41121 Modena, Italy; marco.pappalardo@unimore.it; 4 Nuclear Medicine Unit, Department of Oncohaematology, Fondazione PTV Policlinico Tor Vergata University Hospital, 00133 Roma, Italy; luca.filippi@ptvonline.it; 5 Department of Nuclear Medicine, “Santa Maria Goretti” Hospital, 04100 Latina, Italy

**Keywords:** breast cancer, radioembolization, resin, glass, objective response rate, disease control rate, survival, prognosis

## Abstract

Aim: To meta-analyze the utility of transarterial radioembolization (TARE) in patients with liver metastatic breast cancer (BC), based on the objective response rate (ORR) and disease control rate (DCR). Methods: A literature search was performed retrieving studies with (1) at least 10 patients with liver metastatic BC treated with TARE and (2) adequate information to derive ORR and DCR. The ORR is the ratio between patients with liver lesions showing complete response (CR) or partial response (PR) over the total number of patients treated with TARE; the DCR is the ratio between patients with CR, PR, or stable disease (SD) over the total number of patients treated with TARE. Results: Eighteen studies (650 patients) were eligible; the ORR of TARE resulted 50.71% (95% C.I.: 40.04–61.36) and the DCR resulted 88.37% (95% C.I.: 81.89–93.57). Taking into account resin spheres (395 patients), the ORR was 60.35% (95% C.I.: 46.55–73.36) and the DCR was 92.73% (95% C.I.: 87.17–96.80%). Considering glass spheres (144 patients), the ORR was 32.38% (95% C.I.: 18.43–48.16) and the DCR was 82.69% (95% C.I.: 59.29–97.26). Conclusions: This meta-analysis favors the use of TARE in patients with liver metastatic BC either with resin or glass spheres.

## 1. Introduction

Breast cancer (BC) is the most common cause of oncological death in women worldwide [1]. BC is a major issue for public health, with liver metastases from BC being a prominent clinical concern, affecting a considerable number of patients with advanced stages of the disease. According to epidemiological data, between 50 and 70% of individuals with metastatic BC will develop liver metastases over the course of their disease [2]. After bones, the liver is the second most typical location for distant metastases in BC patients.

Particularly, patients with HER2-positive and triple-negative (TN) breast tumors have an increased prevalence of hepatic metastases. The liver’s intricate vasculature and vital activities pose significant challenges for treating liver metastases, which are frequently associated with a worse prognosis [3]. Furthermore, the presence of liver metastases can lead to debilitating symptoms, such as jaundice and ascites. Therefore, hepatic dysfunction caused by liver metastases can result in severe morbidity, highlighting the need for efficient management strategies [1,4].

Patients with liver lesions may have the option of hepatic resection as a treatment; however, most of the time, the disease is incurable at the time liver metastases are identified. For liver-only disease, a number of liver-targeted therapies have been tested, with the main goals being palliative care and prolonging survival. Stereotactic body irradiation, transarterial chemoembolization, and radiofrequency and microwave ablation are some of these treatments [5]. It is challenging to treat patients despite the availability of numerous therapeutic alternatives, especially with large liver metastases that are resistant to treatment [5,6]. For patients with BC who have hepatic metastases, transarterial radioembolization (TARE) has emerged as a potentially effective treatment strategy. TARE targets liver cancers directly while preserving healthy tissue by carefully delivering radioactive beads, called microspheres, into the hepatic vessels that supply the liver [3]. The microspheres enter the body and become lodged in the tiny blood vessels close to the tumor. The cancer cells are then eliminated by the radiation from the beads, causing the least amount of harm to the surrounding healthy liver tissue. This therapy aids in tumor growth control and shrinkage [7]. Currently, in oncology, the tumor objective response rate (ORR) and disease control rate (DCR) are critical indicators of therapeutic efficacy and function as crucial endpoints for reporting clinical trial results and are essential to clinical decision-making in everyday practice [8,9]. Significant improvements in the outcome of patients with metastatic BC, particularly in terms of ORR and DCR, have been shown to be possible with TARE. Indeed, TARE can significantly reduce tumor size and increase ORR and DCR. The meta-analysis of Aarts et al. evidences better response rates for TARE and TACE compared to chemo-infusion in chemorefractory liver metastatic BC patients [4]. Additionally, Chang et al. demonstrated that TARE was better tolerated than transarterial chemoembolization (TACE) in patients with liver-dominant BC metastasis, comparing a group of 17 patients receiving TACE (32 treatments) and 30 patients receiving TARE (49 treatments) [10]. Patients with liver metastases from BC frequently have fewer treatment options and a worse prognosis; TARE has the potential to help manage tumor development and alleviate symptoms, which may contribute to an improved quality of life and potentially extend longevity by enhancing ORR and DCR [10]. The main aim of this study was to meta-analyze the clinical utility of TARE in patients with liver metastatic BC, based on the average ORR and DCR. As a secondary aim, we assessed the correlation of the receptor status with ORR and DCR.

## 2. Materials and Methods

The PRISMA standards (Preferred Reporting Items for Systematic Reviews and Meta-Analyses) were followed in the conduct of this meta-analysis [11]. A protocol was developed and kept in an internal department database (No. 012024) before the literature search, outlining the study topic, search tactics, inclusion criteria, quality assessment, data extraction, and statistical analysis.

### 2.1. Literature Search and Inclusion Criteria

A systematic review of the literature was carried out on 30 April 2024 using the CENTRAL and PubMed/MEDLINE databases. “(“Breast Neoplasms”[Mesh] OR Breast[tiab] OR mamma*[tiab]) AND (“Infusions, Intra-Arterial”[MeSH] OR intra arterial infusion*[tiab] OR “radioemboliz*” OR “yttrium”) NOT (Review[pt] OR “Case Reports”[pt])” was the search string used in Pubmed/MEDLINE. In CENTRAL, the query “breast AND (yttrium OR radioembolization)” was utilized. There was no time restriction. Non-English language articles were not included. Every reference that was found was exported to a reference management program (Endnote v. X7.5, Clarivate Analytics).

### 2.2. Study Selection

Two investigators (N.Q., S.I.) reviewed independently the titles and abstracts of the retrieved entries and selected only original articles. Non-original articles and duplicates were excluded. For the primary aim of the meta-analysis (the calculation of the average ORR and DCR), the full text of the remaining articles was then examined by four researchers to ensure they satisfied the following inclusion criteria: (1) at least 10 liver metastatic BC patients who had TARE; (2) sufficient data on response assessment to determine ORR and DCR; and (3) no prior history of cancer in the patients. Articles with fewer than ten patients were excluded in order to lessen the small-study effect—which can be caused by methodological flaws, outcome reporting bias, and clinical heterogeneity. For the secondary aim, information on hormone receptor status was required as additional inclusion criteria. Additionally, the obtained publications’ references were examined for any relevant studies that may have been missed in the systematic search.

### 2.3. Data Extraction

From each article, the following data were extracted: name of authors, publication year, journal, country, study design, type of microspheres (resin, glass, or both), mean activity infused (expressed in Gigabequerel), number of patients, number of patients with extrahepatic disease, mean age, BC type according to the hormone receptor status, assessment criteria for response evaluation, number of patients with response assessment, the median follow-up in months from TARE to the date of last clinical visit or death, and the median overall survival (OS). For each article, the number of patients with complete response (CR), partial response (PR), stable disease (SD), and progressive disease (PD) was extracted, as defined according to response evaluation criteria in solid tumors (RECIST version 1.1) or PET response criteria in solid tumors (PERCIST) [12,13]. CR, PR, SD, and PD were used to calculate ORR and DCR for each study.

### 2.4. Methodological Quality Assessment

The methodological quality of the studies included in the meta-analysis was assessed by an investigator (N.Q.) using the “Quality Assessment of Diagnostic Accuracy Studies” tool, v. 2 (QUADAS-2). The QUADAS-2 tool is designed to evaluate the risk of bias and applicability in diagnostic accuracy studies across four domains: patient selection, index test, reference standard, and flow and timing. Each domain includes signaling questions to guide judgments on risk of bias, while the first three domains also assess concerns regarding applicability [14].

### 2.5. Statistical Analysis

Statistical analysis was carried out using MedCalc Statistical Software version 19.1.3 (MedCalc Software, Ostend, Belgium; https://www.medcalc.org 2020, accessed on 30 May 2024). A statistical analysis was performed on a per-patient basis; the treatment response of liver metastatic lesions was determined based on the response criteria adopted in each article. ORR was calculated as the ratio between the number of patients with CR or PR over the total number of patients treated with TARE; the DCR was calculated as the ratio between the number of patients with CR, PR, or SD over the total number of patients treated with TARE. The pooled ORR and DCR (on a per-patient basis) across the studies were calculated with 95% confidence intervals (CIs) for all the patients, patients receiving glass spheres and patients receiving resin spheres. The I^2^ statistic was used to measure the degree of inconsistency across the studies, with values of 25%, 50%, and 75% representing thresholds for low, moderate, and high heterogeneity. Interpretation of heterogeneity was carried out at a significance level of *p* = 0.05. A random-effects model was used for statistical pooling. For studies with available information on BC type, the correlation between receptor status positivity and ORR and DCR was calculated. The correlation was assessed by calculating the Pearson correlation coefficient between the percentage of patients with ER+, PR+, or HER2+ in each study and the corresponding ORR and DCR values, with a significance level set at a *p* value of 0.05.

## 3. Results

### 3.1. Literature Search

**Figure 1:**
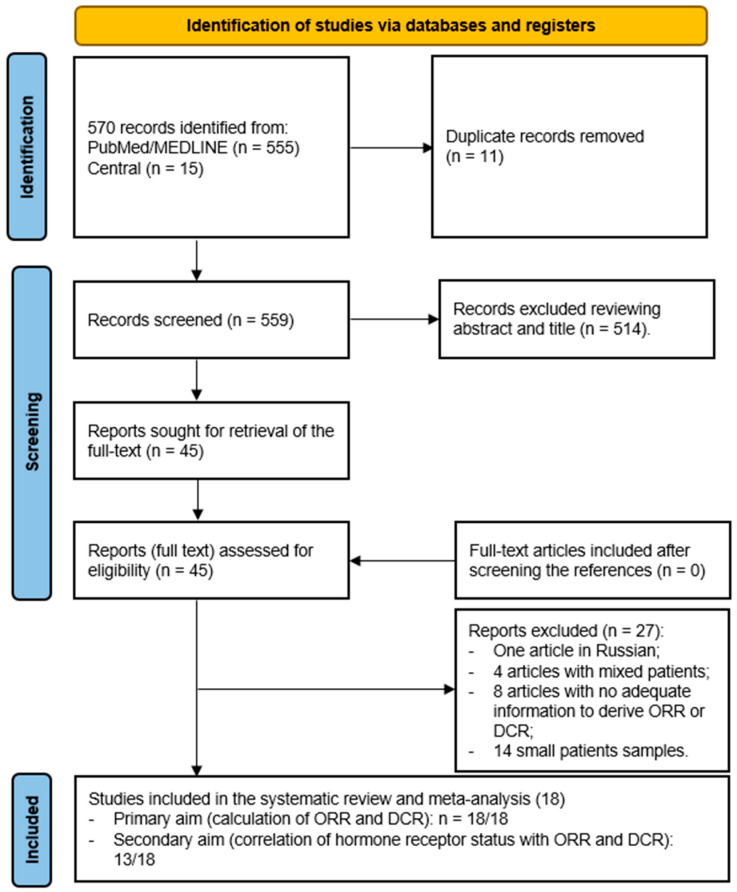
Flow-chart of the literature search.

The comprehensive literature search revealed a total of 570 entries (Figure 1). Eleven entries were excluded as duplicates in the reference manager software. Reviewing titles and abstracts, 514/559 articles were excluded because they were non-original articles or appeared out of the scope of this meta-analysis. The full text of the remaining 45 studies was evaluated to assess the inclusion criteria. No additional records were retrieved after crosschecking the references of the full-text articles. Furthermore, 19 out of 45 articles were excluded due to a patient sample < 10 patients (*n* = 14) or mixed patients without the possibility to discriminate against BC patients (*n* = 4), no sufficient information to calculate the ORR or DCR (*n* = 8) or Russian language (*n* = 1), leading to 18 full-text articles to be eligible for the principal aim of the meta-analysis [3,6,10,15,16,17,18,19,20,21,22,23,24,25,26,27,28,29]. Ten out of eighteen studies included resin spheres, four studies used glass spheres, and the remaining studies used both types of spheres in their patient cohorts. The main characteristics of the studies are reported in Table 1. With respect to the secondary aims of the meta-analysis, among the 18 full-text articles, 5 did not report sufficient information on hormone receptor status, leading to 13 articles.

### 3.2. Qualitative Analysis of the Studies

**Figure 2:**
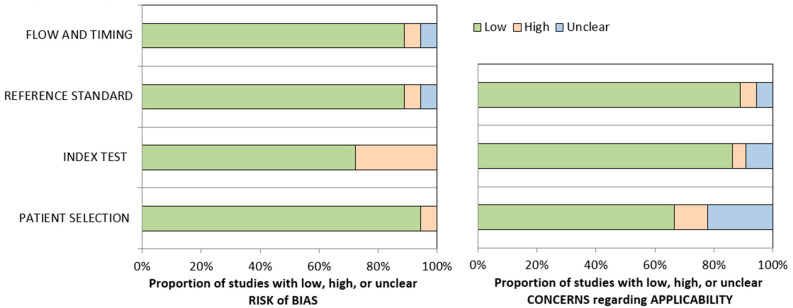
QUADAS 2 assessment results for the included studies of the meta-analysis, indicating low (green color), high (orange color), or unclear (blue color) risk of bias for the relevant domains, and low, high, and unclear concern regarding applicability.

The articles included in the meta-analysis were single-center investigations, published from 2003 to 2021 in the USA (*n* = 7), Germany (*n* = 7), Italy (*n* = 2), the Netherlands (*n* = 1), and Australia (*n* = 1). Five studies were prospective, and 13 studies were retrospective. The patient samples ranged from 14 to 93 patients. Fifteen out of eighteen studies used RECIST (Response Evaluation Criteria in Solid Tumors) to assess the response to therapy; three studies used PERCIST (PET Response Criteria in Solid Tumors). The median follow-up from TARE to the date of last visit ranged from 4 months until death. The median OS ranged from 8 to 35.4 months. Notably, the three studies that used PERCIST criteria reported some of the highest DCRs (100%, 100%, and 96.67%) among all the selected papers (Table 2). Overall, the risk of bias was low across all studies (Figure 2).

### 3.3. Meta-Analysis

**Figure 3:**
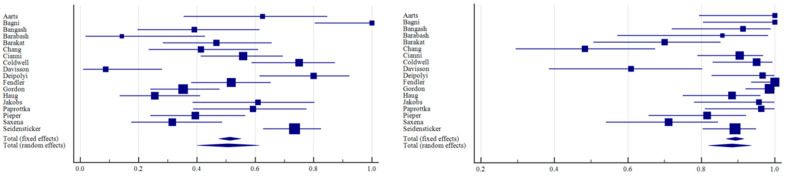
Forest plots of individual studies and pooled ORR (**left**) and DCR (**right**) of patients undergoing TARE.

**Figure 4:**
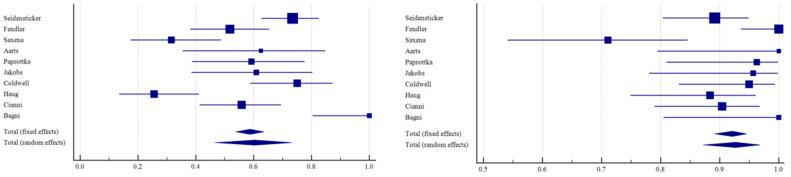
Forest plots of individual studies and pooled ORR (**left**) and DCR (**right**) of patients undergoing TARE with resin spheres.

**Figure 5:**
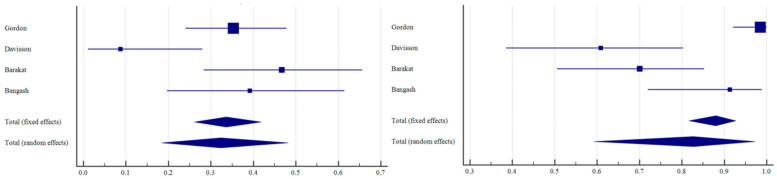
Forest plots of individual studies and pooled ORR (**left**) and DCR (**right**) of patients undergoing TARE with glass spheres.

The number of patients with CR, PR, SD, and PD across all the studies, along with the median follow-up and median OS, is reported in Table 2. In all patient groups (*n* = 650), the ORR resulted in 50.71% (95% C.I.: 40.04–61.36; I^2^: 87%); the DCR resulted in 88.37% (95% C.I.: 81.89–93.57; I^2^: 82%; Figure 3). Taking into account only resin spheres (395 patients), the ORR was 60.35% (95% C.I.: 46.55–73.36; I^2^: 87%) and the DCR was 92.73% (95% C.I.: 87.17–96.80; I^2^: 71%; Figure 4). Considering glass spheres (144 patients), the ORR was 32.38% (95% C.I.: 18.43–48.16; I^2^: 72%) and the DCR was 82.69% (95% C.I.: 59.29–97.26; I^2^: 90%; Figure 5). No significant correlation was found between BC hormone receptor status with ORR and DCR (*p* > 0.05).

## 4. Discussion

TARE has emerged as a valuable therapeutic option for managing liver-dominant metastatic BC. This treatment is especially important for patients whose liver metastases are resistant to traditional systemic therapies, such as chemotherapy. By directly targeting liver tumors with radioactive microspheres, TARE allows for localized high-dose radiation while minimizing exposure to healthy liver tissue, offering a more focused approach compared to systemic treatments [4]. Many studies have shown that TARE can significantly improve the ORR and DCR, which are key indicators of tumor shrinkage and disease stabilization, respectively. Furthermore, TARE has been linked to reduced side effects and improved tolerance when compared to other liver-targeting therapies such as transarterial chemoembolization (TACE). TARE is an essential strategy in the management of advanced-stage disease for patients with liver-dominant metastatic BC since it not only helps control tumor progression and alleviate symptoms but also enhances overall quality of life and extends survival [5].

First, Bangash et al. demonstrated Yttrium-90 (^90^Y) TARE to be a feasible treatment for BC liver metastases in patients whose disease has progressed after standard of care. The authors assessed 27 female patients with progressing liver metastases from BC, despite standard polychemotherapy, in a phase 2 open-label trial. The patients were treated with lobar TARE. Post-treatment was evaluated by (a) tumor response via computed tomography (CT) and/or positron emission tomography (PET); (b) biochemical toxicity; and (c) survival outcomes. At 90-day follow-up CT, tumor response showed: (a) complete or partial response in 9 patients (39.1%); (b) stable disease in 12 patients (52.1%); and (c) disease progression in 2 patients (8.8%). PET scans revealed positive tumor responses in 17 patients (63%). Only three patients (11%) experienced grade 3 bilirubin toxicity, linked to disease progression [16]. Other four prospective studies were included in the present meta-analysis [6,15,17,19]. In particular, Aarts et al. assessed the safety and feasibility of intra-arterial mitomycin C (MMC) infusion after TARE using ^90^Y resin microspheres in liver metastatic breast cancer (LMBC) patients. Using MRI, PET/CT, and laboratory testing, the therapy response was assessed after 6–8 weeks. MMC infusion was given in escalating dose cohorts A (6 mg), B (12 mg), C (24 mg), and D (up to 72 mg) if there was no advancement in the illness. After every two cycles, if there was no progression, Cohort D would obtain MMC. Four patients were not able to receive MMC infusion because of extrahepatic illness or instability, leaving sixteen patients suitable for ^90^Y microspheres. The MMC dose was modified in four patients from Cohort D as a result of either illness progression or biochemical toxicity. Following ^90^Y treatment, there was only one grade 3 adverse event—a gastrointestinal ulcer that required prolonged hospitalization. In conclusion, in 75% of patients, sequential MMC infusion after TARE was possible, although care should be taken to prevent side effects, including reflux, after ^90^Y treatment [6]. The safety of TARE is confirmed also in the prospective study of Coldwell and coworkers, in a total of 44 women, with a median follow-up of 14 months (1–42 months). Indeed, the authors did not document any treatment-related procedure deaths or radiation related veno-occlusive liver failures. Interestingly, computed tomographic imaging partial response was 47% and positron emission tomographic response was 95% [19].

The only researchers to evaluate TACE with TARE for treating breast cancer with liver metastases were Chang et al. [10]. They found that TARE had fewer side events (44 vs. 71%) and a longer median survival time (MST) (12.9 vs. 4.9 months). To verify these findings, additional randomized controlled studies are required. When treating liver metastases that are incurable, external beam radiation therapy (EBRT) has historically been employed more often than TARE. Stereotactic body radiation treatment (SBRT) has a 1-year overall survival (OS) rate of 21–85% for breast cancer that has metastasized to the liver. According to PERCIST standards, local control rates were 100% and 80% at one and two years, respectively. In this context, EBRT, including SBRT as a specialized approach, has several limitations in the treatment of liver metastases compared to TARE, including respiratory motion, the requirement for fiducial markers, increased radiation exposure to healthy liver tissue, and the need for repeated therapeutic sessions [30].

In patients with liver metastatic BC, the current meta-analysis demonstrates a favorable response to TARE based on the pooled ORR and DCR, which are crucial markers of therapeutic efficacy in clinical research. The majority of the studies (13 out of 18) were retrospective, indicating that the data utilized were taken from pre-existing records. This could lead to bias or restrictions because of inadequate data or differences in therapeutic practices.

However, the selected had robust methodology, according to the QUADAS-2 assessment tool. The included studies exhibited significant variability, as indicated by the high heterogeneity (I^2^ = 87% for ORR and 82% for DCR in the overall patient group). This variability may be attributed to variations in patient populations, treatment procedures, or follow-up methodologies. The confidence in deriving general inferences from the pooled results may be diminished due to this unpredictability, even though the confidence intervals provide a range where the genuine effects may fall. We also divided the results according to the kinds of spheres that were employed. The high ORR and DCR values for resin spheres indicate that this treatment may be superior to others in terms of disease control. There is even more heterogeneity in the DCR and a lower ORR for glass spheres than for resin spheres, suggesting that patient responses to this treatment are more variable. This could imply that, in comparison to resin spheres, glass spheres may not be as consistently beneficial across various patient groups or clinical situations. However, compared to patients receiving resin spheres, the group of patients receiving TARE with glass spheres is much too small to make firm results.

There was no significant relationship between ORR or DCR and hormone receptor status, according to the data. Treatment decisions and prognostic factors for hormone-sensitive malignancies (such as breast cancer) are frequently heavily influenced by the hormone receptor status of the patient.

The lack of a correlation implies that the hormone receptor status of the cancer may not have a significant impact on the treatment’s efficacy. This may be a noteworthy discovery since it suggests that the treatment may be broadly relevant to various cancer subtypes; however, more research is required to fully investigate this aspect.

There were certain limitations on our meta-analysis. Small sample sizes were provided by the selected studies. Owing to the heterogeneous sample size of the majority of the studies, it was not feasible to conduct a subgroup analysis based on the histological type of BC. In addition, it should be emphasized that a limitation of our study is the method of assessing treatment response, which was performed in three cases according to PERCIST criteria and in the remaining cases according to RECIST. Furthermore, the category ‘partial response’ inherently includes variability in the depth and degree of response. Finally, it should be noted that, although our meta-analysis shows the potential of TARE to achieve satisfying disease control in mBC, higher ORR and BCR do not automatically translate into benefits in terms of OS or PFS. These topics should be the subject of future investigations.

Further studies, ideally prospective and involving larger cohorts, are needed to better define the efficacy and safety of TARE in the clinical setting of hepatic metastases from breast cancer. These studies should also consider the differences between the various types of microspheres and the varying intervals of post-TARE radiological surveillance.

## 5. Conclusions

The present meta-analysis favors the use of TARE in patients with liver metastatic BC. In patients with BC, TARE provides good ORR and DCR either with resin or glass spheres.

## Figures and Tables

**Table 1:** Main characteristics of the studies included in the meta-analysis.

Authors	Year	Journal	Country	Study Design (P/R)	Type of Microspheres (R/G)	Mean Activity Infused (GBq)	Number of Patients	Number of Patients with Extrahepatic Disease	Mean Age (Years)	BC Type (Hormone Receptor Status)
Barakat [3]	2022	*Adv Radiat Oncol*	USA	R	G	NR	31	21	65.5	ER+: 25; PR: +21; HER2+: 5
Seidensticker [29]	2021	*Cancers*	Germany	R	R	NR	93	69	group 1 (*n* = 59): 56; group 2 (*n* = 34): 55	ER+: 73; PR+: 61; HER2+: 16; TN: 14
Aarts [6]	2020	*Radiol Oncol*	The Netherlands	P	R	1.68	16	9	59	ER+: 8; PR+: 8; HER2+: 0
Davisson [20]	2020	*J Vasc Interv Radiol*	USA	R	G	2	24	18	57	ER+: 20; PR+: 12; HER2+: 2
Deipolyi [21]	2020	*Cardiovasc Intervent Radiol*	USA	R	R/G	2.4	30	30	51.5	ER+: 24; PR+: 20; HER2+: 7
Barabash [17]	2018	*Radiology*	Germany	P	R/G	1.2	14	7	60	NR
Chang [10]	2018	*Anticancer Res*	USA	R	R/G	0.79	30	20	55	ER+: 21; PR+: 20; HER2+: 2
Pieper [27]	2016	*J Vasc Interv Radiol*	Germany	R	R/G	1.35	44	NR	56.1	ER+/PR+: 20; ER+/PR−: 6; ER−/PR−: 1
Fendler [22]	2016	*J Nucl Med*	Germany	R	R	1.6	81	54	61	ER+: 60; PR+: 40; Her2+: 28
Bagni [15]	2015	*Cancer Biother Radiopharm*	Italy	P	R	1.8	17	10	59.2	ER+: 15; PR+: 13; HER2+: NR
Saxena [28]	2014	*Ann Surg Oncol*	Australia	R	R	1.67 ± 0.36 (0.79–2.38)	40	24	54.4	NR
Gordon [23]	2014	*J Vasc Interv Radiol*	USA	R	G	1.52	75	58	54.4	ER+/PR+: 46; ER−/PR−: 46; HER2+: 18; HER2-: 37; TN: 5; unknown: 20
Cianni [18]	2013	*Eur Radiol*	Italy	R	R	1.9	52	24	57.5	NR
Haug [24]	2012	*J Nucl Med*	Germany	R	R	1.7	58	38	58	ER+: 45; PR+: 37; Her2+: 23
Paprottka [26]	2011	*Cardiovasc Intervent Radiol*	Germany	R	R	2.08 (median); range: 1.7–2.5	30	17	58	low/negative hormone receptor expression: 6
Jakobs [25]	2008	*J Vasc Interv Radiol*	Germany	R	R	1.9	30	17	58	HER2+: 6
Bangash [16]	2007	*J Vasc Interv Radiol*	USA	P	G	1.7	27	0	52	NR
Coldwell [19]	2007	*Int J Radiation Oncology Biol Phys*	USA	P	R	2.1	44	29	58	ER+: 31; HER2+: 12

P: prospective; R: retrospective; R: resin; G: glass; ER: estrogen receptor; PR: progesterone receptor; HER2: human epidermal growth factor receptor 2; TN: triple negative; NR: not reported.

**Table 2:** List of the studies included in the meta-analysis with corresponding response rates. ECOG: eastern cooperative oncology group status.

Authors	Assessment Criteria for Response Evaluation	Percentage of Patients with ORR	Percentage of Patients with DCR	CR	PR	SD	PD	Total Patients with Response Assessments	Median Follow-Up (Months) from TARE to Date of Last Visit/Death	Median OS (Months)
Barakat	RECIST	46.67	70.00	1	13	7	9	30	12	13
Seidensticker	RECIST	73.49	89.16	2	59	13	9	83	until death	8
Aarts	RECIST	62.50	100.00	0	10	6	0	16	until death	12.6
Davisson	RECIST	8.70	60.87	0	2	12	9	23	22.3	35.4
Deipolyi	PERCIST	80.00	96.67	7	17	5	1	30	until death	15
Barabash	RECIST	14.29	85.71	0	2	10	2	14	until death	9
Chang	RECIST	41.38	48.28	0	12	2	15	29	9	12.9
Pieper	RECIST	39.47	81.58	0	15	16	7	38	4	6.1
Fendler	mPERCIST	52.00	100.00	0	29	27	0	56	until death	8
Bagni	PERCIST	100.00	100.00	2	15	0	0	17	until death	13.5
Saxena	RECIST	31.58	71.05	2	10	15	11	38	11.2	13.6
Gordon	RECIST	35.30	98.50	0	24	43	1	68	until death or last follow-up visit	6.6
Cianni	RECIST	55.77	90.38	0	29	18	5	52	until death	11.5
Haug	RECIST. WHO	25.60	88.40	0	11	27	5	43	6.4	10.9
Paprottka	RECIST	59.26	96.30	0	16	10	1	27	14.2	NR
Jakobs	RECIST	60.87	95.65	0	14	8	1	23	4.2	11.7
Bangash	RECIST	39.13	91.30		9	12	2	23	until death	ECOG 0 (*n* = 15): 6.8; ECOG 1–3 (*n* = 12): 2.6
Coldwell	RECIST	75.00	95.00	7	23	8	2	40	14	14
